# The RNA-binding protein LUC7L2 mediates MITA/STING intron retention to negatively regulate innate antiviral response

**DOI:** 10.1038/s41421-021-00277-y

**Published:** 2021-06-22

**Authors:** Chen Li, Lu Feng, Wei-Wei Luo, Cao-Qi Lei, Mi Li, Hong-Bing Shu

**Affiliations:** 1 Department of Infectious Diseases, Zhongnan Hospital of Wuhan University, College of Life Sciences, Frontier Science Center for Immunology and Metabolism, Medical Research Institute, Research Unit of Innate Immune and Inflammatory Diseases of Chinese Academy of Medical Sciences, Wuhan University, Wuhan, Hubei China; 2 Wuhan Institute of Virology, Chinese Academy of Sciences, Hubei, Wuhan China

**Keywords:** Innate immunity, Cell signalling

## Abstract

MITA (also known as STING) is an ER-located adaptor protein, which mediates DNA-triggered innate immune response and is critically involved in autoimmune diseases and tumorigenesis. MITA is regulated by post-translational modifications, but how post-transcriptional mechanisms are involved in the regulation of MITA is still largely unknown. Here, we identified the RNA-binding protein LUC7L2 as a negative regulator of DNA virus-triggered innate immune response. LUC7L2-deficient mice exhibited resistance to lethal herpes simplex virus 1 (HSV-1) infection and reduced HSV-1 loads in the brain. Mechanistically, LUC7L2 directly bound to intron 3 of MITA precursor messenger RNA, inhibited its splicing and promoted its nonsense-mediated decay, leading to its downregulation at protein level. LUC7L2-deficient cells had markedly increased MITA level, leading to heightened innate antiviral response. Finally, LUC7L2 was induced following HSV-1 infection. Our findings reveal a feedback negative post-transcriptional regulatory mechanism for regulation of MITA-mediated innate immune response to viral and aberrant cellular DNA.

## Introduction

The innate immune system acts as the first line of host defense against microbial infection. Upon microbial infection, cellular pattern recognition receptors (PRRs) recognize structurally conserved microbial components called pathogen-associated molecular patterns (PAMPs), which triggers a series of signaling events that lead to induction of type I interferons (IFNs), pro-inflammatory cytokines and other downstream innate immune effectors. These downstream effectors mediate the inhibition of microbial replication, clearance of infected cells and facilitation of adaptive immune response^1,2^.

Cytosolic DNA, derived from invaded DNA pathogens or mislocated cellular and mitochondrial DNA, acts as a major type of PAMPs to initiate innate immune response. Cytosolic DNA is sensed by the cyclic GMP-AMP (cGAMP) synthase (cGAS), which utilizes GTP and ATP as substrates to synthesize cGAMP. cGAMP acts as a second messenger, which binds to the ER-located adaptor protein MITA (Mediator of IRF3 Activation), also known as STING (Stimulator of Interferon Genes)^3–6^. Upon binding to cGAMP, MITA undergoes oligomerization and translocates from ER via ER-Golgi intermediate compartments (ERGIC) and Golgi apparatus to perinuclear punctate structures. During the trafficking processes, MITA recruits the kinases TBK1 and IKK, leading to activation of the transcription factors IRF3 and NF-κB as well as induction of downstream effector genes^2,7–9^.

The structural configuration, protein level, activity, and cellular localization of MITA are regulated by co-factors and various post-translational modifications, such as phosphorylation, polyubiquitination, and sumoylation^2,9–18^. It has been shown that transcription of MITA is induced by type I IFNs^19^, and LSm14a is important for maintaining mature MITA mRNA level in dendritic cells (DCs)^20^.

Most multi-exon genes (~95%) have more than one alternative splice forms due to exon skipping/inclusion, alternative 3′ and 5′ splice site selection, or intron retention (IR)^21^. IR has emerged as a previously under-appreciated mechanism of post-transcriptional regulations. Unlike the other two alternative splicing events, IR rarely contributes to proteomic diversity^22^. However, IR normally acts as a negative regulatory mechanism of gene expression by slowing down splicing kinetics^23^, increasing potential nuclear degradation by nuclear exosomes, and increasing potential cytoplasmic degradation by nonsense-mediated decay (NMD)^24^. It has been demonstrated that IR plays an important role in regulating gene expression in a wide range of processes including cellular differentiation^25,26^ and tumorigenesis^27^. In addition, wide-spread IR throughout mouse and human cell and tissue types suggests that IR events adjust the transcriptome of a cell^28^. However, with few exceptions, the factors that control IR events and thus potentially shape gene expression programs of cells remain enigmatic.

In this study, we identified the putative RNA-binding protein LUC7L2 as a negative regulator of MITA-mediated innate immune response. LUC7L2-deficiency results in strengthened innate immune response to DNA virus or synthetic DNA in vitro and in vivo. Mechanistically, LUC7L2 binds to and results in the retention of intron 3 of *MITA* gene. LUC7L2-deficiency causes increased *MITA* mRNA and protein levels, which mediates enhanced innate immune response. Moreover, LUC7L2 is induced following DNA virus infection. Our findings suggest that LUC7L2-mediated MITA IR represents an important feedback negative regulatory mechanism of innate immune response to cytosolic DNA.

## Results

### LUC7L2 negatively regulates innate immune response to DNA virus

**Fig. 1:**
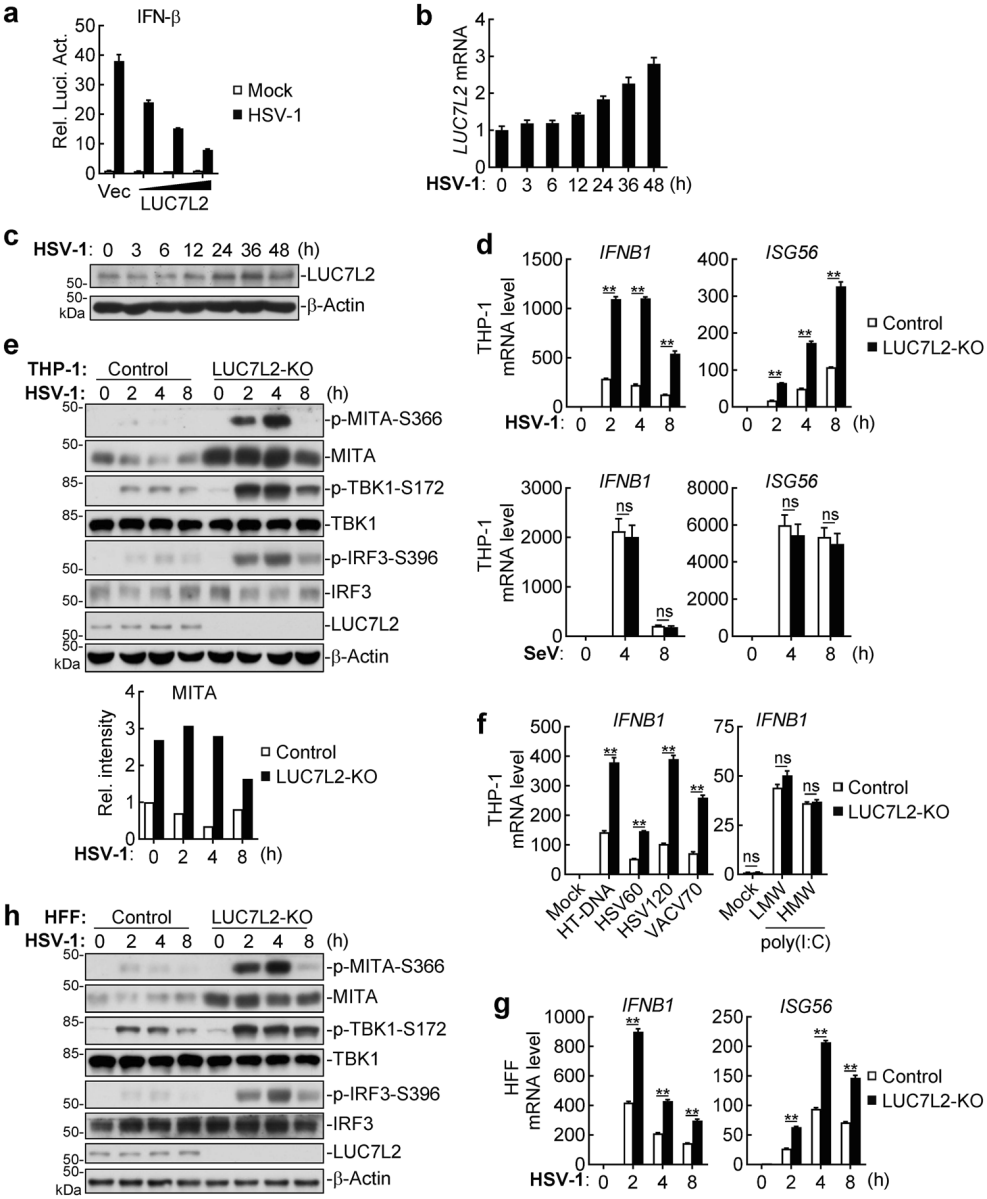
LUC7L2 negatively regulates DNA virus-triggered signaling. **a** LUC7L2 inhibits HSV-1-induced activation of the *IFNB1* promoter in a dose-dependent manner. HeLa cells (5 × 10^5^) were transfected with *IFNB1* promoter luciferase reporter (100 ng) and increased amounts of LUC7L2 plasmid. Twenty hours later, the cells were infected with HSV-1 (MOI = 1) or left uninfected for 12 h before reporter assays. **b**, **c** HSV-1 infection induces expression of LUC7L2. THP-1 cells were infected with HSV-1 (MOI = 1) for the indicated time before qPCR analysis of LUC7L2 mRNA level (**b**) or immunoblotting analysis of LUC7L2 protein level (**c**). **d** Effects of LUC7L2-deficiency on virus-induced transcription of downstream antiviral genes. LUC7L2-deficient and control THP-1 cells were left untreated or infected with HSV-1 or SeV (MOI = 1, respectively) for the indicated time before qPCR analysis. **e** Effects of LUC7L2-deficiency on HSV-1-induced phosphorylation of signaling components. LUC7L2-deficient and control THP-1 cells were left uninfected or infected with HSV-1 (MOI = 1) for the indicated time before immunoblotting analysis with the indicated antibodies. The lower graph shows the relative intensities of MITA proteins, which were quantitated by densitometry using ImageJ and normalized to β-actin levels. **f** Effects of LUC7L2-deficiency on transcription of *IFNB1* gene induced by transfected synthetic nucleic acids. LUC7L2-deficient and control THP-1 cells (1 × 10^6^) were mock-transfected or transfected with the indicated DNA and RNA (0.5 μg each) for 4 h before qPCR analysis. **g** Effects of LUC7L2-deficiency on HSV-1-induced transcription of antiviral genes in HFFs. LUC7L2-deficient and control HFFs were left uninfected or infected with HSV-1 (MOI = 1) for the indicated time before qPCR analysis. **h** Effects of LUC7L2-deficiency on HSV-1-induced phosphorylation of signaling components in HFFs. LUC7L2-deficient and control HFFs were left uninfected or infected with HSV-1 (MOI = 1) for the indicated time before immunoblotting analysis with the indicated antibodies. Data shown in **a**, **b**, **d**, **f**, and **g** are means ± SEM from one representative experiment performed in triplicates. **P < 0.01 (unpaired *t*-test); ns not significant.

We performed expression screens of ~15,000 independent human cDNA clones for their abilities to regulate IFN-β promoter activity. LUC7L2 was identified as a candidate clone that inhibited HSV-1-induced activation of the IFN-β promoter in a dose-dependent manner (Fig. 1a). Additionally, we found that LUC7L2 was induced by HSV-1 at both mRNA and protein levels in monocytic THP-1 cells (Fig. 1b, c), further suggesting a potential role of LUC7L2 in innate immune response to DNA virus.

To determine whether endogenous LUC7L2 inhibits DNA virus-triggered signaling, we constructed LUC7L2-deficient human monocytic THP-1 cells by CRISPR/Cas9-mediated gene editing. Knockout of LUC7L2 significantly potentiated transcription of downstream antiviral genes such as *IFNB1* and *ISG56* induced by infection of the DNA virus HSV-1 but not the RNA virus Sendai virus (SeV) (Fig. 1d). Consistently, phosphorylation of MITA, TBK1 and IRF3 induced by HSV-1 infection, which are hallmarks of virus-triggered signaling, was markedly enhanced in LUC7L2-deficient THP-1 cells (Fig. 1e). These results suggest LUC7L2 plays an important role in innate immune response to DNA virus.

We next investigated whether LUC7L2 is involved in dsDNA-induced transcription of downstream antiviral genes. It has been shown that transfected dsDNAs, such as Herring testis DNA (HT-DNA), the 60-mer and 120-mer dsDNA representing the genome of HSV-1 (HSV60 and HSV120), and 70-mer dsDNA representing vaccinia virus (VACV) genome (VACV70) efficiently induce transcription of antiviral genes^6,29–31^. We found that deficiency of LUC7L2 potentiated transcription of the *IFNB1* gene induced by transfection of these synthetic dsDNAs but not the dsRNA analog poly(I:C) in THP-1 cells (Fig. 1f). In similar experiments, transcription of *IFNB1* and *ISG56* genes (Fig. 1g) and phosphorylation of MITA, TBK1 and IRF3 (Fig. 1h) induced by HSV-1 in LUC7L2-deficient human foreskin fibroblasts (HFFs) were also markedly increased compared with control cells. Collectively, these data suggest that LUC7L2 plays an important role in DNA virus- and cytosolic dsDNA-triggered induction of downstream antiviral genes.

### LUC7L2 inhibits antiviral responses in vivo

**Fig. 2:**
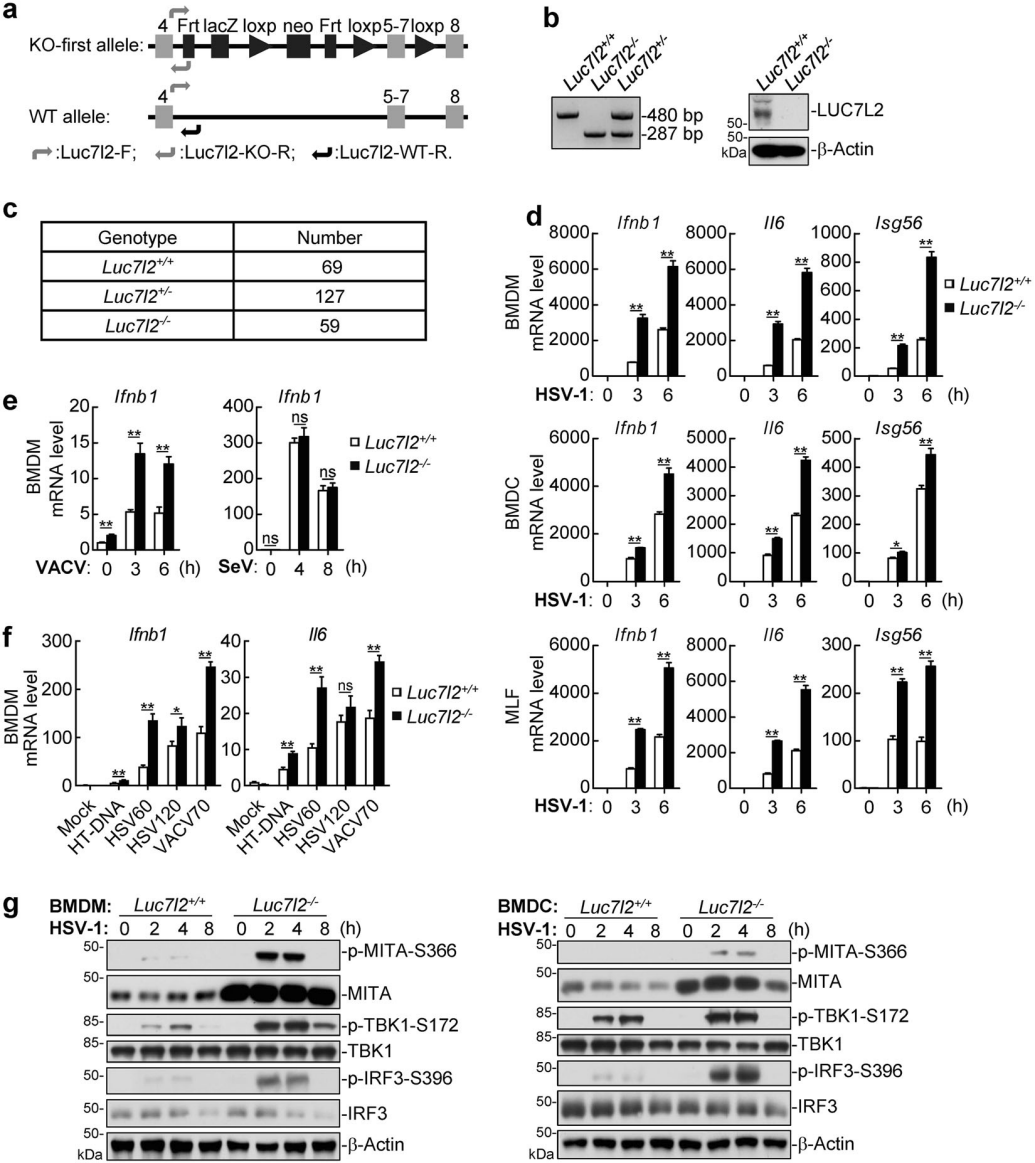
LUC7L2-deficiency promotes innate immune response to DNA virus in primary mouse cells. **a** A schematic presentation of the targeting strategy for *Luc7l2*-knockout mice. **b** Genotyping of Luc7l2-knockout mice by PCR (left panel). BMDMs from wild-type and Luc7l2-knockout mice were analyzed by immunoblots with the indicated antibodies (right blots). **c** Genotypes of the offspring from the breeding of *Luc7l2*^*+/−*^ mice. **d** Effects of LUC7L2-deficiency on HSV-1-induced transcription of antiviral genes. *Luc7l2*^*+/+*^ and *Luc7l2*^*−/−*^ BMDMs, BMDCs or MLFs were left uninfected or infected with HSV-1 (MOI = 1) for the indicated time before qPCR analysis. **e** Effects of LUC7L2-deficiency on virus-induced transcription of *IFNB1* gene. *Luc7l2*^*+/+*^ and *Luc7l2*^*−/−*^ BMDMs were left uninfected or infected with VACV or SeV (MOI = 1, respectively) for the indicated time before qPCR analysis. **f** Effects of LUC7L2-deficiency on transcription of antiviral genes induced by transfected dsDNAs. *Luc7l2*^*+/+*^ and *Luc7l2*^*−/−*^ BMDMs (5 × 10^5^) were mock-transfected or transfected with the indicated DNA (0.5 μg each) for 4 h before qPCR. **g** Effects of LUC7L2-deficiency on HSV-1-induced phosphorylation of signaling components. *Luc7l2*^*+/+*^ and *Luc7l2*^−*/−*^ BMDMs or BMDCs were left uninfected or infected with HSV-1 (MOI = 1) for the indicated time before immunoblotting analysis with the indicated antibodies. Data shown in **d**–**f** are means ± SEM from one representative experiment performed in triplicates. *P < 0.05, **P < 0.01 (unpaired *t*-test); ns not significant.

To further elucidate the function of LUC7L2 in vivo, we obtained LUC7L2-deficient mice by the knockout-first strategy (Fig. 2a). The successful targeting of *Luc7l2* gene was verified by genotyping and immunoblotting analysis (Fig. 2b). *Luc7l2*^*−/−*^ mice were bred normally and genotypes of offspring matched the Mendelian ratio (Fig. 2c). To investigate the functions of LUC7L2, we generated bone marrow-derived macrophages (BMDMs), bone marrow-derived dendritic cells (BMDCs) and murine lung fibroblasts (MLFs) from sex- and age-matched wild-type and LUC7L2-deficient mice. qPCR analysis indicated that transcription of *Ifnb1*, *Il6*, and *Isg56* genes induced by HSV-1 was markedly enhanced in LUC7L2-deficient BMDMs, BMDCs and MLFs compared to their wild-type counterparts (Fig. 2d). In similar experiments, transcription of *Ifnb1* gene induced by the DNA virus VACV was also enhanced in LUC7L2-deficient BMDMs compared to wild-type cells, whereas transcription of *Ifnb1* gene induced by SeV was comparable between *Luc7l2*^−*/*−^ and wild-type BMDMs (Fig. 2e). Consistently, transcription of *Ifnb1* and *Il6* genes in response to transfected HT-DNA, HSV60, HSV120, and VACV70 was increased in LUC7L2-deficient BMDMs compared to the wild-type cells (Fig. 2f). Further experiments indicated that deficiency of LUC7L2 enhanced phosphorylation of MITA, TBK1, and IRF3 induced by HSV-1 in BMDMs and BMDCs (Fig. 2g). These results suggest that LUC7L2 specifically regulates viral DNA- but not RNA-triggered innate immune responses in primary mouse cells.

**Fig. 3:**
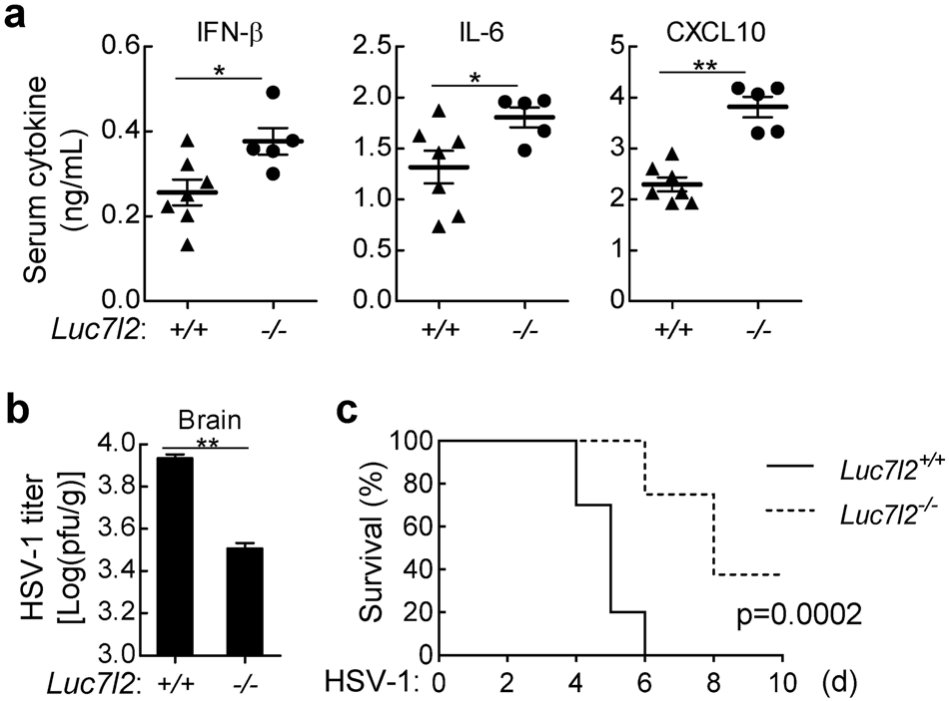
LUC7L2 negatively regulates host defense to DNA virus in mice. **a** Effects of LUC7L2-deficiency on serum levels of IFN-β, IL-6, and CXCL10 induced by HSV-1. *Luc7l2*^*+/+*^ (*n* = 7) and *Luc7l2*^*−/−*^ mice (*n* = 5) (8 weeks old) were intravenously infected with HSV-1 (1 × 10^7^ PFU per mouse) for 3 h before measurements of the indicated serum cytokines by ELISA. **b** Measurements of viral titers in the brain. *Luc7l2*^*+/+*^ and *Luc7l2*^*−/−*^ mice (*n* = 3 per strain, 8 weeks old) were intranasally infected with HSV-1 at 1 × 10^7^ PFU per mouse for 7 days. HSV-1 viral titers in the brains of infected mice were quantified by plaque assays. **c** Effects of LUC7L2-deficiency on HSV-1-induced death of mice. *Luc7l2*^*+/+*^ (*n* = 10) and *Luc7l2*^*−/−*^ mice (*n* = 8) (8 weeks old) were intravenously injected with HSV-1 (1 × 10^7^ PFU per mouse), and the survival rates of mice were monitored daily. *P* = 0.0002 (log-rank test).

To investigate the functions of LUC7L2 in host defense against viral infection in vivo, sex- and age-matched *Luc7l2*^*+/+*^ and *Luc7l2*^*−/−*^ mice were infected with HSV-1. HSV-1-induced IFN-β, IL-6 and CXCL10 production in the sera of *Luc7l2*^*−/−*^ mice was significantly increased compared to wild-type mice (Fig. 3a). Given that HSV-1 is a neurotropic virus that causes sporadic encephalitis, we intranasally infected *Luc7l2*^*+/+*^ and *Luc7l2*^*−/−*^ mice with HSV-1 and examined HSV-1 loads in the brains. We found that the replication of HSV-1 was suppressed in the brains from *Luc7l2*^*−/−*^ mice compared to those from *Luc7l2*^*+/+*^ mice on day 7 post HSV-1 infection (Fig. 3b). Additionally, *Luc7l2*^*−/−*^ mice exhibited a later onset of death and a higher survival rate compared to wild-type mice (Fig. 3c). These data suggest that LUC7L2 negatively regulates innate immune response in responses to DNA virus in vivo.

### LUC7L2 downregulates MITA level by mediating its intron retention

**Fig. 4:**
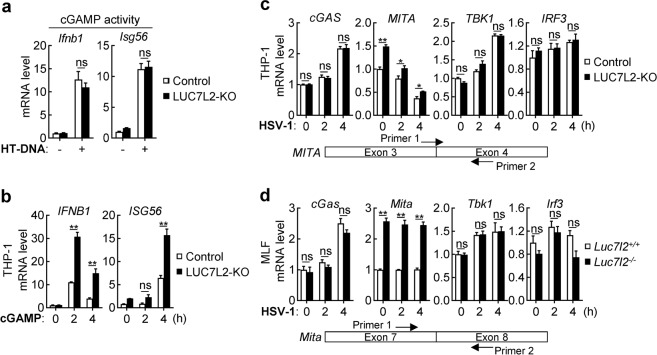
LUC7L2 inhibits MITA level. **a** Effects of LUC7L2-deficiency on cGAMP synthesis induced by transfected HT-DNA. Control or LUC7L2-deficient THP-1 cells (5 × 10^7^) were transfected with HT-DNA (10 μg) for 4 h. The heat-inactivated extracts were delivered to digitonin-permeabilized RAW264.7 cells. Induction of *Ifnb1* and *Isg56* genes was then measured by qPCR analysis. **b** Effects of LUC7L2-deficiency on transcription of antiviral genes induced by cGAMP. LUC7L2-deficient and control THP-1 cells (1 × 10^6^) were left untreated or treated with 2′3′-cGAMP (0.2 μg/mL) for the indicated time before qPCR analysis. **c**, **d** Effects of LUC7L2-deficiency on mRNA levels of the indicated signaling components. LUC7L2-deficient and control THP-1 (**c**) or MLF (**d**) cells were left uninfected or infected with HSV-1 (MOI = 1) for the indicated time before qPCR analysis. The locations of qPCR primers for MITA are shown. Data shown are means ± SEM from one representative experiment performed in triplicates. *P < 0.05, **P < 0.01 (unpaired *t*-test); ns, not significant.

To investigate the mechanisms on how LUC7L2 is involved in DNA-triggered signaling, we first examined cGAMP activity in HT-DNA-transfected wild-type and LUC7L2-deficient THP-1. The results indicated that extracts from HT-DNA-transfected wild-type and LUC7L2-deficient THP-1 exhibited comparable cGAMP activity (Fig. 4a). We next determined whether LUC7L2 inhibited cGAMP-triggered downstream signaling. We found that cGAMP-induced transcription of *IFNB1* and *ISG56* genes was enhanced in LUC7L2-deficient THP-1 compared to control cells (Fig. 4b). Additionally, our earlier data showed that phosphorylation of MITA, TBK1, and IRF3 induced by HSV-1 was also enhanced in LUC7L2-deficient human and mouse cells compared with control cells (Figs. 1e, h, 2g). These results suggest that LUC7L2 regulates a component between cGAMP and TBK1. Interestingly, we noted that the protein level of MITA/STING, but not other examined components including TBK1 and IRF3, was markedly upregulated in LUC7L2-deficient THP-1 cells (Figs. 1e, h, 2g). All these data suggest LUC7L2 functions by regulating MITA level.

**Fig. 5:**
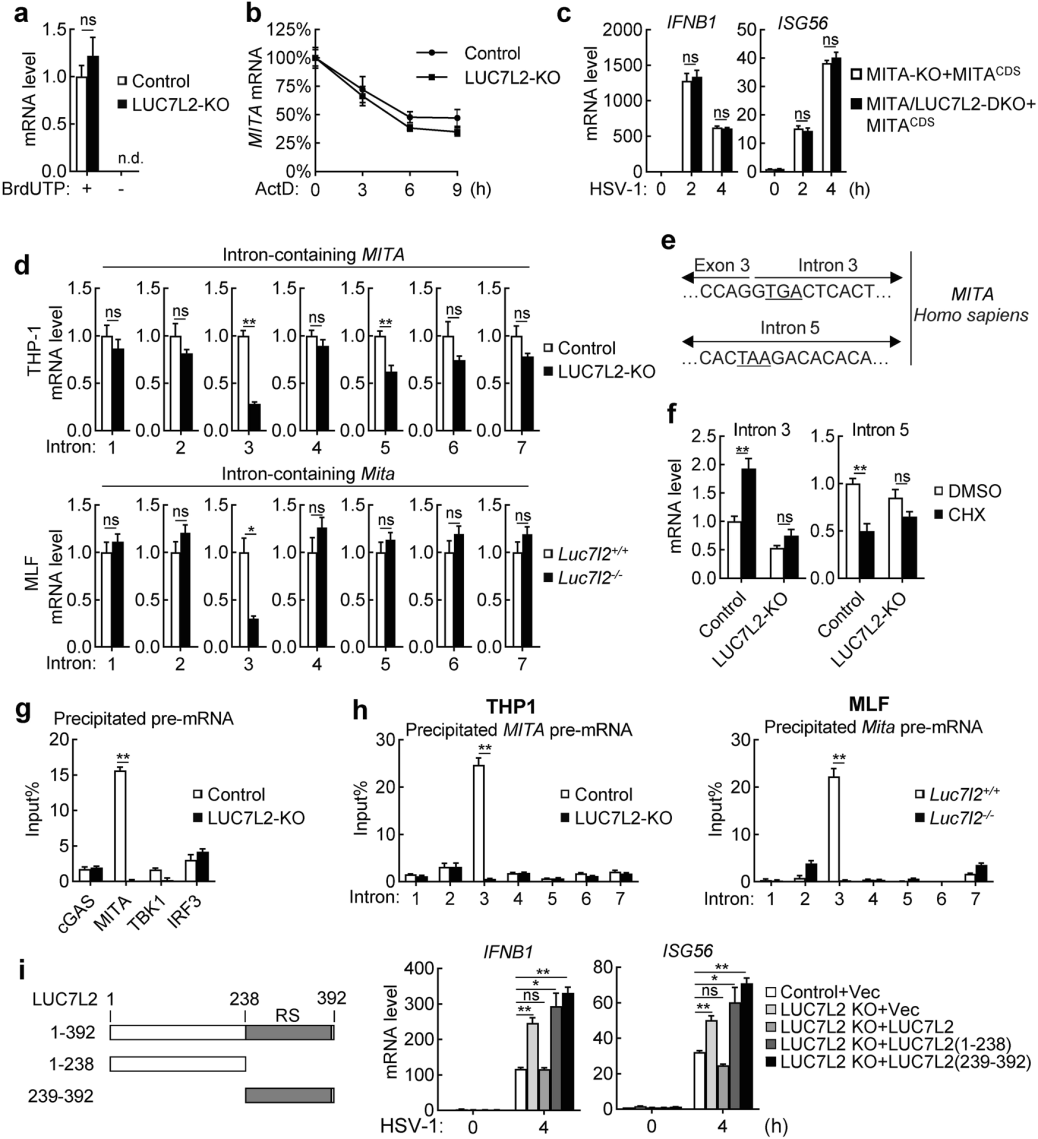
LUC7L2 promotes MITA intron retention. **a** Effects of LUC7L2-deficiency on transcriptional activity of *MITA/STING* gene. Crude nuclei from control and LUC7L2-deficient THP-1 cells were isolated for NRO assays. n.d., not detectable. **b** Effects of LUC7L2-deficiency on MITA mRNA stability. Control and LUC7L2-deficient THP-1 cells (1 × 10^6^) were treated with Act D (5 μg/mL) for the indicated time before qPCR analysis. **c** Effects of LUC7L2-deficiency on HSV-1-induced transcription of antiviral genes in MITA cDNA-reconstituted cells. MITA-deficient or MITA and LUC7L2 double-deficient THP-1 cells reconstituted with an expression plasmid for *MITA-* coding cDNA (MITA^CDS^) were uninfected or infected with HSV-1 (MOI = 1) for the indicated time before qPCR analysis. **d** Effects of LUC7L2-deficiency on levels of MITA transcript containing retained introns. MITA transcripts containing retained introns from LUC7L2-deficient and control human THP-1 or MLF cells were measured by qPCR using primers matching to the indicated intron and exon next to it. **e** Partial sequences of intron 3 and 5 of MITA (*Homo sapiens)* showing the PTCs. **f** Effects of LUC7L2-deficiency on CHX-induced increase of MITA transcripts containing retained introns. LUC7L2-deficient and control THP-1 cells (1 × 10^6^) were treated with DMSO or CHX (30 μg/mL) for 6 h before qPCR analysis. **g** LUC7L2 binds to MITA pre-mRNA. Nuclei from LUC7L2-deficient and control THP-1 cells were isolated and lysed for RIP assays with LUC7L2 antibody. The percentage of the precipitated RNA to input RNA of the indicated genes was calculated based on qPCR results. **h** LUC7L2 binds to intron 3 of MITA transcripts. LUC7L2-deficient and control human THP-1 cells or MLFs were treated with UV (254 nm, 400 mJ/cm^2^). Nuclei from LUC7L2-deficient and control cells were isolated and lysed for RIP assays with LUC7L2 antibody. The percentage of the precipitated MITA pre-mRNA containing the indicated introns to input RNA of the indicated genes was calculated based on qPCR results. **i** Effects of reconstitution of LUC7L2 and its truncation mutants on HSV-1-induced transcription of downstream genes in LUC7L2-deficient THP-1 cells. LUC7L2-deficient THP-1 cells were reconstituted with wild-type LUC7L2 or its truncation mutants by retroviral-mediated gene transfer. The reconstituted THP-1 cells were left uninfected or infected with HSV-1 (MOI = 1) for 4 h before qPCR analysis. A schematic presentation of LUC7L2 and its truncation mutants is shown on the left. Data shown (except **e**) are means ± SEM from one representative experiment performed in triplicates. *P < 0.05, **P < 0.01 (unpaired *t*-test); ns, not significant.

We next investigated how LUC7L2 regulates MITA level. We found that the mRNA level of *MITA* but not other examined genes including *cGAS*, *TBK1*, and *IRF3* was upregulated in LUC7L2-deficient human THP-1 cells and MLFs (Fig. 4c, d), suggesting that LUC7L2 specifically downregulates MITA mRNA level. We next performed nuclear run-on (NRO) assays to measure the transcriptional activity of *MITA* gene via quantification of biochemically labeled nascent RNA derived from isolated nuclei. As shown in Fig. 5a, nascent transcripts of *MITA* gene were comparable between LUC7L2-deficient and control THP-1 cells, suggesting that LUC7L2 does not regulate the transcription of *MITA* gene.

We next examined whether the increase of mature MITA mRNA level in LUC7L2-deficient cells is a result of its slower degradation. Quantitative evaluation of the mRNA decay rate revealed a half-life of 5.5 h for MITA mRNA in LUC7L2-deficient THP-1 cells and 4.9 h in control cells, respectively (Fig. 5b). These results suggest that LUC7L2 does not regulate MITA mRNA stability. In addition, we reconstituted MITA-deficient THP-1 cells with an expression plasmid of MITA cDNA (only containing the coding sequence) (THP-1-MITA^CDS^). qPCR analysis showed that LUC7L2-deficiency had no marked effects on HSV-1-induced transcription of *IFNB1* and *ISG56* in the reconstituted THP-1-MITA^CDS^ cells (Fig. 5c). These results suggest that LUC7L2 regulates a step at MITA pre-mRNA splicing.

We next measured the level of MITA transcripts containing the unspliced introns in LUC7L2-deficient and control human THP-1 or MLFs by qPCR using primers matching to the individual intron and the exon next to it. We found that the level of MITA transcripts containing intron 3 was decreased in both LUC7L2-deficient THP-1 cells and MLFs, whereas the level of MITA transcripts containing intron 5 was decreased only in LUC7L2-deficient THP-1 cells (Fig. 5d). The levels of MITA transcripts containing other examined introns were comparable between LUC7L2-deficient and control cells (Fig. 5d). Sequence analysis indicated that intron 3 and 5 of MITA in *Homo sapiens* contain premature termination codons (PTCs) (Fig. 5e), which often lead to rapid degradation of the transcripts by the NMD pathway^32^. Cycloheximide (CHX) is reported to be an inhibitor of NMD^33^. CHX treatment enhanced the level of intron 3- but not 5-containing MITA transcripts (Fig. 5f), indicating that retention of intron 3 in MITA transcripts promotes their degradation by the NMD pathway. In these experiments, CHX treatment did not affect intron 3-containing MITA/STING transcripts in LUC7L2-deficient cells (Fig. 5f). Native RNA immunoprecipitation (RIP) showed that LUC7L2 bound to pre-mRNA of MITA but minimally to pre-mRNA of other examined components including cGAS, TBK1 and IRF3 (Fig. 5g). We next performed cross-linked RIP, which precisely maps the binding region of LUC7L2 to MITA transcripts in human THP-1 or MLFs. The results confirmed that LUC7L2 bound to intron 3 of MITA/STING pre-mRNA in both human and mouse cells (Fig. 5h). We further investigated whether intron retention of MITA by LUC7L2 is dependent on its RNA-binding capacity. It has been shown that LUC7L2 binds to RNA via its C-terminal Arg/Ser-rich domain (RS domain)^34^. Reconstitution experiments indicated that wild-type LUC7L2 but not its N- or C-terminal truncation mutants inhibited HSV-1-induced transcription of *IFNB1* and *ISG56* genes in THP-1 cells (Fig. 5i), suggesting that an intact LUC7L2 is important for its inhibition of MITA level and virus-induced innate immune signaling. Taken together, these results suggest that LUC7L2 promotes retention of intron 3 in MITA/STING transcripts, which leads to their NMD-dependent degradation and regulation of MITA protein level.

## Discussion

MITA/STING is an adaptor protein that plays critical roles in innate immune response to viral DNA or aberrant cellular/mitochondrial DNA. The protein level and activity of MITA are delicately regulated under physiological conditions and during infection to ensure the optimal activation and timely termination of innate immune responses. Several host factors have been reported to regulate MITA via post-translational modifications^2,9–18^, but how MITA is regulated at the post-transcriptional level remains enigmatic. In this study, we found that LUC7L2 was induced following viral infection and promoted MITA/STING intron retention, leading to decreased expression of MITA/STING and attenuation of innate immune responses to DNA virus.

Several lines of evidence suggest that LUC7L2 plays an important role in innate immune responses to DNA virus. Deficiency of LUC7L2 potentiated HSV-1-induced transcription of downstream antiviral genes in various cell types. The serum cytokines such as IFN-β, IL-6, and CXCL10 induced by HSV-1 were increased in *Luc7l2*^*−/−*^ in comparison to *Luc7l2*^*+/+*^ mice. The viral loads of HSV-1 in the brains were markedly decreased in *Luc7l2*^*−/−*^ mice in comparison to their wild-type counterparts after HSV-1 infection. LUC7L2-deficient mice also exhibited resistance to lethal HSV-1 infection.

Mechanistic studies suggest that LUC7L2 promotes MITA/STING intron retention. Analysis of the DNA-triggered signaling events indicated that LUC7L2 regulated MITA but not other components in innate immune signaling. Further experiments indicated that the protein and mRNA levels of MITA were upregulated in LUC7L2-deficient cells. mRNA decay assays indicated that MITA mRNA in LUC7L2-deficient THP-1 cells was as stable as that in wild-type cells. NRO assays showed that LUC7L2-deficiency had no marked effects on transcription of *MITA* gene. The simplest explanation from these results is that LUC7L2 plays an important role in regulating MITA pre-mRNA processing. In support of this hypothesis, qPCR experiments showed that unspliced intron 3 level of MITA transcripts was markedly downregulated in LUC7L2-deficient THP-1 cells, implying a role for LUC7L2 in intron retention. CHX treatment enhanced level of MITA transcripts containing intron 3, suggesting that intron 3-containing MITA transcripts are degraded by the NMD pathway. RIP assays showed that LUC7L2 specifically bound to intron 3 of MITA pre-mRNA. Consistently, the level of MITA transcripts containing intron 3 was decreased in LUC7L2-deficient THP-1 cells. The sequences of human and mouse MITA intron 3 share ~65% identity with a couple of identical sequence stretches (data not shown). Similarly, our results indicated that the level of mouse Mita transcripts containing intron 3 was decreased in LUC7L2-deficient MLFs, and mouse LUC7L2 bound to mouse Mita intron 3. These results suggest that LUC7L2 regulates MITA level in human and mouse cells with similar mechanisms.

Based on our results, we conclude that LUC7L2 promotes intron 3 retention of MITA pre-mRNA, which contributes to both retention of the MITA pre-mRNA in the nucleus and its NMD-dependent degradation, and leads to subsequent downregulation of MITA protein level. Since knockout of LUC7L2 increases MITA level in the absence of viral infection, our results suggest that LUC7L2 regulates MITA level constitutively in the cell. It is possible that keeping MITA level at a proper level in the cell is an important mechanism for avoiding MITA-mediated autoimmune activity. Since LUC7L2 is transcriptionally induced upon DNA virus infection, our findings suggest that LUC7L2-mediated MITA intron 3 retention also represents an important feedback negative regulatory mechanism of innate immune response to viral or aberrant cellular DNA. Recently, various studies have demonstrated that regulation of MITA activity by small molecular compounds may provide strategies for immunotherapy of cancers or intervention of autoimmune diseases^35^. It would be interesting to investigate potential roles of LUC7L2 in regulation of carcinogenesis, cancer immunotherapy, and autoimmune diseases.

## Materials and methods

### Reagents, antibodies, cells, and viruses

GM-CSF (PeproTech); 2’3’-cGAMP (InvivoGen); Cycloheximide, Tween-80, and digitonin (Sigma-Aldrich); Lipofectamine 2000, Opti-MEM, M-MLV Reverse Transcriptase (Invitrogen); RiboLock RNase Inhibitor (Thermo Scientific); Protease Inhibitor Cocktail (MCE); polybrene (Millipore); RNAiso Plus (TaKaRa); SYBR Green mix (Bio-Rad); RPMI 1640 Medium, Dulbecco’s Modified Eagle Medium (Gibco); Dual-Specific Luciferase Assay Kit (Promega); ELISA kits for murine IFN-β (PBL), murine CXCL10 (Neobioscience Technology), and murine IL-6 (BioLegend); mouse antibodies against β-actin (Sigma-Aldrich); rabbit antibodies against phospho-MITA (S366), MITA and phospho-IRF3 (S396) (Cell Signaling Technology), phospho-TBK1 (S172) and TBK1 (Abcam), BrdU (Santa Cruz Biotechnology), and LUC7L2 (ABclonal) were purchased from the indicated manufacturers. Mouse antisera against murine IRF3 was raised using the full-length recombinant protein as antigen. HEK293T cells were originally provided by Dr. G. Johnson (National Jewish Health, Denver, CO). THP-1 cells were obtained from the American Type Culture Collection. HFFs were provided by Dr. Min-Hua Luo (Wuhan Institute of Virology, CAS). SeV (Cantell strain) (Charles River Laboratories), VACV (Tian-Tan Strain), and HSV-1 (KOS strain) (China Center for Type Culture Collection, Wuhan, China) were obtained from the indicated resources.

### Animals

All animal experiments were performed in accordance with the Wuhan University Animal Care and Use Committee guidelines.

### *Luc7l2*-knockout mice and genotyping

*Luc7l2*-knockout mice on the C57BL/6 background were purchased from CAM-SU GRC. Genotyping by PCR was performed using the following primers:

Luc7l2-Forward: 5’-CCAGGAAGCAGAAGTGAGTGGGTTG-3’;

Luc7l2-WT-Reverse: 5’-GGCTCAGCAGTTAAGATTACAGGCT-3’;

Luc7l2-KO-Reverse: 5’-CTCCTACATAGTTGGCAGTGTTTGG-3’.

The mice were bred in specific pathogen-free facilities at Wuhan University Medical Research Institute. Six to eight-week-old mice were used in the experiments and littermates were used as controls. Experiments were conducted without blinding, with age- and sex-matched mice.

### Constructs

Expression plasmids for LUC7L2 and MITA were constructed into pMSCV-PuroR plasmid by standard molecular biology techniques. Guide-RNA plasmids targeting *LUC7L2* were constructed into lentiCRISPR V2 vector (provided by Dr. Shu-Wen Wu, Wuhan University).

### Expression screens

The cDNA expression clones encoding ∼15,000 independent human cDNAs (CCSB-Board lentiviral expression library) were purchased from GE Healthcare. The clones were individually transfected into HeLa cells together with the IFN-β promoter reporter plasmid. Twenty hours later, cells were left untreated or infected with HSV-1 (MOI = 1) for 12 h before reporter assays.

### Preparation of primary mouse cells

For preparation of BMDMs, mouse bone marrow-derived monocytes (5 × 10^6^) were cultured in 100-mm dishes in 5 mL 10% M-CSF-containing conditional medium from L929 cells for 3–5 days. For preparation of BMDCs, mouse bone marrow-derived monocytes (5 × 10^6^) were cultured in medium containing murine GM-CSF (50 ng/mL) for 6–8 days. For preparation of lung fibroblasts, lungs were minced and digested in calcium and magnesium free HBSS containing 10 μg/mL type II collagenase and 20 μg/mL DNase I for 1 h at 37 °C with shaking. Cell suspension was centrifuged at 448× *g* for 5 min. The cells were then plated in culture medium (1:1 (v/v) DMEM/Ham’s F-12 containing 10% FBS, 50 U/mL penicillin, 50 μg/mL streptomycin, 15 mM HEPES, 2 mM _L_-glutamine).

### DNA oligonucleotides

Sequences of the synthetic DNAs, including VACV70, HSV60, and HSV120 have been described^30^. The sequence of oligonucleotides used to construct the LUC7L2 gRNA plasmid is 5’-CAGAGACTTACCGTCCCGGG-3’.

### qPCR

Total RNA was isolated for qPCR analysis to measure mRNA levels of the indicated genes according to the manufacturer’s protocol (TaKaRa). Data shown are the relative abundance of the indicated mRNA normalized to that of *ACTB* gene. Sequences of gene-specific primer pairs were as followed:

murine *Ifnb1*, 5′-TCCTGCTGTGCTTCTCCACCACA-3′ and 5′-AAGTCCGCCCTGTAGGTGAGGTT-3′;

murine *Isg56*, 5′-ACAGCAACCATGGGAGAGAATGCTG-3′ and 5′-ACGTAGGCCAGGAGGTTGTGCAT-3′;

murine *Il6*, 5′-TCTGCAAGAGACTTCCATCCAGTTGC-3′ and 5′-AGCCTCCGACTTGTGAAGTGGT-3′;

murine *cGas*, 5′-CATGTGAAGATTTCTGCTCCT-3′ and 5′-TCACAAGATAGAAAGCACCTG-3′;

murine *Mita*, 5′-AAATAACTGCCGCCTCATTG-3′ and 5′-TGGGAGAGGCTGATCCATAC-3′;

murine *Tbk1*, 5′-GAGAAGACTGTGAAAGTGTATGAG-3′ and 5′-GTGTATGTCTGAAATCTCACCC-3′;

murine *Irf3*, 5′-CTACACTCTGTGGTTCTGC-3′ and 5′-GTAGGAACAACCTTGACCA-3′;

murine *Actb*, 5′-CTCCATCCTGGCCTCACTGTC-3′ and 5′-TCGTACTCCTGCTTGCTGATCC-3′;

murine *Mita* intron 1, 5′-GCTAGGTGTCCACTGGAGTGT-3′ and 5′-GTCACTCACCAGCAGCCACA-3′;

murine *Mita* intron 2, 5′-CCCCAGAAGCATAGCTGTGG-3′ and 5′-GGATGCAGGTTGGAGTATGGC-3′;

murine *Mita* intron 3, 5′-CACCTAGCCTCGCACGAAC-3′ and 5′-CAGAGGGTTACCTGGACTGGAC-3′;

murine *Mita* intron 4, 5′-TCTTCCCACACCACCCACC-3′ and 5′-TCTTCCCACACCACCCACC-3′;

murine *Mita* intron 5, 5′-GGCCTGGTCATACTACATTGGGT-3′and 5′-CCAGAGGTGCCCTACCTGG-3′;

murine *Mita* intron 6, 5′-CCAACAGCGTCTACGAGATTCTG-3′ and 5′-GGATTCAGAGTTCTTGGGAGCTGG-3′;

murine *Mita* intron 7, 5′-CCCGAGTCTCGAAATAACTGCC-3′ and 5′-CTCCAGCTGTATCAGTCTCTTCTTACC-3′;

human *IFNB1*, 5′-TTGTTGAGAACCTCCTGGCT-3′ and 5′-TGACTATGGTCCAGGCACAG-3′;

human *ISG56*, 5′-TCATCAGGTCAAGGATAGTC-3′ and 5′-CCACACTGTATTTGGTGTCTACG-3′;

human *cGAS*, 5′-TGGTGAAAGGGGTTGTGGAC-3′ and 5′-GTGCAGAAATCTTCACGTGCT-3′;

human *MITA*, 5′-CACATCCACTCCAGGTACC-3′ and 5′-AGAAATAGATGGACAGCAGCA-3′;

human *TBK1*, 5′-GGAAACAGTTATTATCGCTGAC-3′ and 5′-AGGCAGAGTTTCTTGTAACTC-3′;

human *IRF3*, 5′-TCTACGAGTTTGTGAACTCAG-3′ and 5′-GAATGTCTTCCTGGGTATCAG-3′;

human *cGAS* pre-mRNA, 5′-GCTTGTGGGTGATTGAGTCAT-3′ and 5′-AACACGCTCCAGTCAGGAAC-3′;

human *MITA* pre-mRNA, 5′-CCATTCTCCATCCGCCTGG-3′ and 5′-GGCGTACTCCAGGACACAG-3′;

human *TBK1* pre-mRNA, 5′-GTGATGTTCAGTCCGCAGA-3′ and 5′-ACCCATAAGCCAGGTAGACG-3′;

human *IRF3* pre-mRNA, 5′-GCCAGAGGTGGTAAACAGTG-3′ and 5′-TTGGTGTCTAATCCCGTGC-3′;

human *MITA* intron 1, 5′-GGAACCCGCTGTTCAGAGCT-3′ and 5′-GCCTGAGTCCTGGGGTCAG-3′;

human *MITA* intron 2, 5′-CATCCAGAGCAGCCAGTGTC-3′ and 5′-CTCGCCTCTGGGTGCCAG-3′;

human *MITA* intron 3, 5′-TCTGCAGCCTGGCTGAGGA-3′ and 5′-AGGGAGTGACACACGTTGGA-3′;

human *MITA* intron 4, 5′-CTCTCGCAGGCACTGAACA-3′ and 5′-CTCAGGGCAGGTCACACCA-3′;

human *MITA* intron 5, 5′-CGGATATCTGCGGCTGATCC-3′ and 5′-CACAAGAGGCTGTGTGTC-3′;

human *MITA* intron 6, 5′-GACCGGTGACCATGCTGG-3′ and 5′-GTCCTGACCCCTCCTCAGAG-3′;

human *MITA* intron 7, 5′-GAGGACATCCTGGCAGATGC-3′ and 5′-TCCTGCCCTCCAGCCTATC-3′;

human *LUC7L2*, 5′-GAGATTAGTGCTGAAGTAGCAGC-3′ and 5′-GCCCGTGCTTTCTCTACTTC-3′;

human *ACTB*, 5′-AAGACCTGTACGCCAACACA-3′ and 5′-AGTACTTGCGCTCAGGAGGA-3′.

### Transfection

HEK293T and HeLa cells were transfected by standard calcium phosphate precipitation method. THP-1 cells and BMDMs were transfected by lipofectamine 2000 with Opti-MEM according to the manufacturer’s instructions.

### Dual-luciferase assay

Luciferase assays were performed using a Dual-Specific Luciferase Assay Kit. To normalize for transfection efficiency, pRL-TK (Renilla luciferase) reporter plasmid (0.01 μg) was added to each transfection. Firefly luciferase activities were normalized based on Renilla luciferase activities.

### Plaque assay

Eight-week-old mice were infected with HSV-1 for 7 days, and the brains of mice were weighed and homogenized for 5 s in PBS. After homogenization, the brain suspensions were centrifuged at 1620× *g* for 30 min, and the supernatants were used for plaque assays on monolayers of Vero cells seeded in 24-well plates. The cells were infected by incubation for 1 h at 37 °C with serial dilutions of brain suspensions. After infection for 1 h, 2% methylcellulose was overlaid, and the plates were incubated for about 48 h. Cells were fixed with 4% paraformaldehyde for 15 min and stained with 1% crystal violet for 30 min before plaque counting.

### ELISA

Eight-week-old *Luc7l2*^*+/+*^ and *Luc7l2*^*−/−*^ mice were intravenously infected with HSV-1 for 3 h, and the sera of mice were collected for measurement of IFN-β, CXCL10, and IL-6 cytokines by ELISA according to the manufacturer’s protocol.

### cGAMP extraction

The indicated THP-1 cells (5 × 10^7^) were left untreated or transfected with HT-DNA (10 μg) for 3 h. Cells were then homogenized by dunce homogenizer in hypotonic buffer (10 mM Tris-HCl, pH 7.4; 10 mM KCl; 1.5 mM MgCl_2_). After centrifugation at 13,000 rpm for 20 min, the supernatant was heated at 95 °C for 10 min and centrifuged at 13,000 rpm for another 10 min to remove denatured proteins. The heat-resistant supernatants containing cGAMP were delivered for digitonin permeabilization assays.

### Digitonin permeabilization

The protocol for digitonin permeabilization was previously described^20^. The cells were treated with 2’3’-cGAMP or cGAMP-containing cellular extracts in digitonin permeabilization solution (50 mM HEPES, pH 7.0; 100 mM KCl; 3 mM MgCl_2_; 0.1 mM DTT; 85 mM Sucrose; 0.2% BSA; 1 mM ATP; 0.1 mM GTP, and 10 μg/mL digitonin) at 37 °C for 30 min. The cells were then incubated in regular medium for the indicated time before qPCR.

### Retroviral-mediated gene transfer

HEK293T cells plated on 100-mm dishes were transfected with pMSCV-PuroR (10 μg) together with the pGag-Pol (10 μg) and pVSV-G (3 μg) plasmids. Two days after transfection the viruses were harvested to infect the indicated cells in the presence of polybrene (8 μg/mL). One day later, the infected cells were selected by puromycin (2 μg/mL for THP-1 cells) for at least 5 days.

### CRISPR-Cas9 knockout

The protocols for gene engineering using the CRISPR-Cas9 system were previously described^36,37^. Briefly, double-stranded oligonucleotides corresponding to the target sequences were cloned into the lentiCRISPR V2 plasmid, which was transfected with packaging plasmids (psPAX2 and pMD2.G) into HEK293T cells. Two days after transfection, the viruses were harvested to infect THP-1 or HFF cells. The infected cells were selected with puromycin (2 μg/mL for THP-1 cells and 0.5 μg/mL for HFFs) for at least 5 days.

### Nuclear run-on assay

The experiments were performed as described previously^38^. Briefly, THP-1 cells (1 × 10^7^) were incubated for 5 min at 4 °C with cold swelling buffer (10 mM Tris-HCl, pH 7.5; 2 mM MgCl_2_; and 3 mM CaCl_2_). After centrifugation at 1000 rpm for 10 min, the pellet was resuspended in lysis buffer (10 mM Tris-HCl, pH 7.5; 2 mM MgCl_2_; 3 mM CaCl_2_; 0.5% NP-40; 10% glycerol; and 2 U/mL RNase inhibitor) and gently pipetted up and down 20 times using a p1000 tip with the end cut off. The nuclei were pelleted at 1000 rpm for 10 min and washed with lysis buffer and then with storage buffer (50 mM Tris-HCl, pH 8.3; 40% glycerol; 5 mM MgCl_2_; 0.1 mM EDTA; and 2 U/mL RNase inhibitor). The nuclei were added to reaction buffer (10 mM Tris-HCl, pH 8.3; 5 mM MgCl_2_; 1 mM DTT; 300 mM KCl; and 100 U/mL RNase inhibitor) containing ATP, CTP, GTP, and Brd-UTP or UTP (500 mM each) in the presence of 0.5% sarkosyl. After transcription at 30 °C for 30 min, nuclear run-on (NRO)-RNAs were extracted using the RNAiso plus reagent, according to the recommendation of manufacturer, and treated with DNase I. The NRO-RNAs were denatured at 70 °C for 10 min and then incubated with preblocked (PBS with 0.1% Tween-20, 0.1% polyvinylpyrrolidone, and 0.1% UltraPure BSA) agarose beads conjugated with anti-BrdU antibodies. After 30 min at room temperature on a rotating platform, beads were washed three times with PBSTR (PBS with 0.1% Tween-20 and 8 U/mL RNase Inhibitor). Immunoprecipitated NRO-RNAs were extracted using the RNAiso plus reagent, and reverse transcription was performed following standard protocol, except that glycogen was added during isopropanol precipitation step, and random primers were used for reverse transcription instead of oligo-dT. qPCR was performed using the following NRO-primers, which were designed for nascent pre-mRNAs: NRO-*MITA*, 5′-GGAAAGAAGTGAGGGAGGGTA-3′ and 5′-TGCCAGAGAATGGAGAGTCC-3′; and NRO-*ACTB*, 5′-TCTCTGACCTGAGTCTCCTTTG-3′ and 5′-CCTACACCCACAACACTGTCTT-3′.

### Native RNA immunoprecipitation (RIP)

The indicated THP-1 cells (1 × 10^7^) were harvested by spun down gently (5 min, 4 °C, 1000× *g*). The cells were rinsed twice with 10 mL of cold PBS followed by spun down gently (5 min, 4 °C, 1000× *g*). The cell pellet was resuspended in 0.1–1 mL of polysome lysis buffer (100 mM KCl, 5 mM MgCl_2_, 10 mM HEPES, pH 7.0, 0.5% NP-40, 1 mM DTT, and 100 U/mL RNase Inhibitor) supplemented with Protease Inhibitor Cocktail. At this point the mRNP lysate, which could be passed through a small gauge needle if necessary, was frozen. Protein-G beads were pretreated at 4 °C with NT2 (50 mM Tris-HCl, pH 7.4, 150 mM NaCl, 1 mM MgCl_2_, and 0.05% NP-40) supplemented with 5% BSA to a final ratio of 1:5 for at least 1 h. Five microgram of antibody per sample was added to 75 μL of protein-G-coated magnetic beads and incubated at 4 °C overnight. Following incubation, the beads were spun down and washed with 1 mL of ice-cold NT2 buffer for five times. Following final wash, the beads were resuspended in 850 μl of NT2 and supplemented with 200 U of RNase inhibitor, 10 μL of 100 mM DTT, and EDTA to 20 mM. Frozen lysate was thawed and centrifuged at 15,000× *g* for 15 min. The cleared supernatant was removed and 100 μL was added to the prepared beads. Input removed at this step. Beads and lysates were incubated for 4 h at 4 °C with mixing. The beads were washed five times with ice-cold NT2 and then resuspended in 100 μL of NT2 buffer supplemented with 30 μg of proteinase K and 1% SDS. The sample was incubated with shaking at 65 °C for 2 h. RNA was isolated by RNAiso plus reagent as the manufacturer’s instructions and reverse transcribed with oligo-dT. Quantification of precipitated pre-mRNA of specific genes was performed by qPCR.

### Cross-linked RNA immunoprecipitation

The indicated cells (1 × 10^7^) were treated with UV (254 nm, 400 mJ/cm^2^) and then harvested by spun down gently (5 min, 4 °C, 1000× *g*). The cells were rinsed twice with 10 mL of cold PBS followed by spun down gently (5 mins, 4 °C, 1000× *g*). Final cell pellet was resuspended in 4 mL of cell lysis buffer (10 mM Tris-HCl, pH 7.4, 10 mM NaCl, 0.5% NP-40, 1 mM DTT, 200 U/mL RNase inhibitor, and EDTA-free Protease Inhibitor Cocktail) and incubated for 15 min on ice to release nuclei. Nuclei were recovered by centrifugation at 1000× *g* for 10 min at 4 °C and then resuspended with 3 mL of nuclei resuspension buffer (50 mM HEPES-NaOH, pH 7, 10 mM MgCl_2_, 1 mM DTT, 200 U/mL RNase inhibitor, and EDTA-free Protease Inhibitor Cocktail). Then nuclei were sonicated to DNA fragments in a range between 200 and 1000 bp. After the sonication, the chromatins were digested with 250 U/mL of DNase for 30 min at 37 °C. The DNase reaction was terminated with 20 mM of EDTA. The chromatin samples were supplemented with 1% Triton X-100, 0.1% sodium deoxycholate, 0.01% SDS, and 140 mM NaCl. Five microgram of antibody per sample was added to 75 μL of protein-G-coated magnetic beads and incubated at 4 °C overnight. Following incubation, beads were spun down and washed with 1 mL of ice-cold nuclei resuspension buffer for five times. Following final wash, the beads were resuspended in 75 μL of nuclei resuspension buffer. The samples were centrifuged at 20,000× *g* for 10 min at 4 °C. The cleared supernatant was removed and 925 μL was added to the prepared antibody-conjugated beads. The samples were incubated overnight at 4 °C with mixing. The beads were washed with 1 mL of immunoprecipitation buffer (150 mM NaCl, 10 mM Tris-HCl, pH 7.4, 1 mM EDTA, 1 mM EGTA, pH 8, 1% TritonX-100, 0.5% NP-40, 1 mM DTT, 200 U/mL RNase inhibitor, and EDTA-free Protease Inhibitor Cocktail) for six times and then resuspended in 100 μL of NT2 buffer supplemented with 30 μg of proteinase K and 1% SDS. The sample was incubated with shaking at 65 °C for 2 h. RNA was isolated by RNAiso plus reagent as the manufacturer’s instructions and reverse transcribed with random primers. Quantification of precipitated specific sites of pre-mRNA of *MITA* was performed by qPCR.

### Statistics

Data were analyzed using a Student’s unpaired *t*-test, multiple *t*-test or two-way ANOVA with GraphPad Prism 8. For the mouse survival study, Kaplan–Meier survival curves were generated and analyzed by log-rank test. The number of asterisks represents the degree of significance with respect to *P*-values, with the latter presented within each figure or figure legend.
